# Nutritional status as a predictive factor for paediatric tuberous sclerosis complex-associated kidney angiomyolipomas: a retrospective analysis

**DOI:** 10.1007/s00431-024-05520-8

**Published:** 2024-03-14

**Authors:** Andrew Limavady, Matko Marlais

**Affiliations:** 1 Great Ormond Street Institute of Child Health, University College London, London, UK; 2 Department of Paediatric Nephrology, Great Ormond Street Hospital, London, UK

**Keywords:** Nutritional status, Tuberous sclerosis complex, Angiomyolipoma, Paediatrics

## Abstract

The purpose of this study is to determine the predictive factors of tuberous sclerosis complex (TSC)-associated kidney disease and its progression in children. Retrospective review of children with TSC in a tertiary children’s hospital was performed. Relevant data were extracted, and Cox proportional hazards regression was used to establish predictors of kidney lesions. Logistic regression was conducted to identify factors predicting chronic kidney disease (CKD) and high-risk angiomyolipomas (above 3 cm). Kidney imaging data were available in 145 children with TSC; of these, 79% (114/145) had abnormal findings. The only significant predictive factor for cyst development was being female (HR = 0.503, 95% CI 0.264–0.956). Being female (HR = 0.505, 95% CI 0.272–0.937) and underweight (HR = 0.092, 95% CI 0.011–0.800) both lowers the risk of having angiomyolipomas, but TSC2 mutations (HR = 2.568, 95% CI 1.101–5.989) and being obese (HR = 2.555, 95%CI 1.243–5.255) increases risks. Ten (12%) of 81 children with kidney function tested demonstrate CKD stages II–V, and only angiomyolipomas above 3 cm predict CKD. Additionally, 13/145 (9%) children had high-risk angiomyolipomas, whereby current age (adjusted odds ratio (aOR) 1.015, 95% CI 1.004–1.026) and being overweight/obese (aOR 7.129, 95% CI 1.940–26.202) were significantly associated with angiomyolipomas above 3 cm.

*Conclusions*: While gender and genotype are known predictors, this study includes the novel finding of nutritional status as a predictor of TSC-associated kidney disease. This study sheds light on a possible complex interplay of hormonal influences, obesity, and kidney angiomyolipomas growth, and further investigations focusing on the impact of nutritional status on TSC-associated kidney disease are warranted.

**Table:** 

**What is Known:** • *Gender and genotype are well-studied predictive factors in TSC kidney disease.*	
**What is New:** • *Nutritional status may influence the development and the progression of kidney lesions in children with TSC and should not be overlooked. * • *Management guidelines of TSC-associated kidney disease can address nutritional aspects. *

**Supplementary Information:**

The online version contains supplementary material available at 10.1007/s00431-024-05520-8.

## Introduction

Tuberous sclerosis complex (TSC) is a tumour-suppressor gene syndrome due to the loss of function in either TSC1 or TSC2 genes, disrupting the mechanistic target of the rapamycin (mTOR) pathway [1]. This disruption promotes the growth of benign tumours across multiple organs, representing the hallmark clinical features of TSC. Notably, TSC-associated kidney disease emerges as the leading cause of morbidity and mortality in adults [2]. This pertains to developing kidney cysts and angiomyolipomas that can replace normal parenchyma and cause subsequent chronic kidney disease (CKD) or haemorrhage [3], particularly angiomyolipomas that grow beyond 3–4 cm (high-risk) [4].

Previous studies on TSC patients consistently reported kidney lesions were typically identified between 9 and 12 years, and their prevalence increases with age [5–7]. TSC exhibits three major kidney phenotypes: kidney angiomyolipoma, cystic kidney disease, and rarely renal cell carcinoma. Kidney angiomyolipoma is the characteristic lesion affecting approximately two-thirds of TSC patients. Besides age, the effect of gender and genetic mutations on the development of angiomyolipoma has often been assessed in association studies. Patients with TSC2 mutations had significantly earlier angiomyolipoma detection (13 vs. 23 years) and showed significantly higher rates of multiple and bilateral angiomyolipomas than those with TSC1 mutations [6]. Additionally, TSC2 patients were more likely to progress to high-risk angiomyolipomas, thus requiring interventions [8]. Unlike age and genetic mutations, the association between gender and kidney angiomyolipoma incidence have garnered conflicting results, with some studies suggesting higher prevalence in females, while others found it more prevalent in males, and some studies showed no gender difference [4]. Hormonal influences of oestrogen and progesterone on angiomyolipoma tumour receptors have been hypothesised for the increased prevalence in females [9].

Studies on non-TSC adult patients with kidney cancer have highlighted obesity as a risk factor for its development [10]. To the best of our knowledge, no studies have analysed nutritional status as a predictor for kidney disease in TSC patients. Considering that malnutrition commonly occurs in children, this study aims to understand the influence of nutritional status in kidney lesions development and determine the predictive factors for TSC-associated kidney disease and the complications.

## Materials and methods

### Study population

We performed a retrospective cohort study of all children diagnosed with TSC who received care at Great Ormond Street Hospital for Children in London between January 2011 and December 2022. Inclusion criteria were (1) patients under 19 years of age at the time of diagnosis and (2) definite genetic or clinical diagnosis of TSC. Patients with insufficient clinical information for inclusion in the cohort (for example, patients who were seen only once for a second opinion and lacked complete information in hospital clinical records), and those without available kidney imaging data on record were excluded.

### Data collection

Medical records of eligible children were reviewed. Relevant patient data, including demographic information (age, gender, weight, and height), genetics, and clinical outcomes (blood and urine testing results, and kidney lesion haemorrhage), were extracted and collated in a standardised, anonymised data collection spreadsheet. Kidney involvement was assessed by analysing imaging reports, specifically capturing data from the initial imaging session when the detection of cysts and angiomyolipomas occurred. Nutritional status was determined by calculating the body mass index (BMI) as the ratio of weight (kg) to the squared height (m^2^) at time of index imaging. Subsequently, the nutritional status was categorised based on the CDC centiles classification: underweight (< 5th centiles), normal weight (5th–< 85th centiles), overweight (85th–< 95th centiles), and obesity (> 95th centiles).

Kidney function was assessed through the estimated glomerular filtration rate (eGFR), calculated using the Schwartz formula: 36.5 multiplied by the height at the time of blood sampling (in centimetres), divided by the creatinine levels at time of imaging (in µmol/L) [11]. An eGFR less than 90 mL/min/1.73m^2^ was considered as indicative of CKD stages II–V.

### Data analysis

All analyses were conducted using SPSS version 28 (IBM Corp., Armonk, NY). Descriptive statistics comprised the number of children within each of the four BMI categories, using the normal weight group as the baseline reference. Baseline characteristics were described using median and interquartile range for continuous variables, and frequencies and percentages for categorical variables. The assessment of data normality was conducted by plotting histograms. Cox proportional hazards regression models were used to establish the predictor variables for kidney lesions development. Variables known or hypothesised to be associated with outcomes (gender, genetic mutation, and nutritional status) were included in the models. The models estimated hazard ratios (HR) with 95% confidence intervals to determine significant kidney lesions development predictors. Univariate and multivariable logistic regression was used to assess the predictors of high-risk angiomyolipomas and CKD. The multivariate logistic regression model only included variables with *p*-values < 0.05 on univariate analysis. Predictors included in the regression model include current age, age at first presentation of angiomyolipomas and cysts, gender, genetic mutation, and overweight/obese. No imputations were made for missing data given the exploratory nature of this study. All statistical tests were two-sided and considered significant at a 5% significance level.

## Results

This analysis included a cohort of 145 children diagnosed with TSC with available kidney imaging data. The median current age was 11.2 (interquartile range: 6.8–16.9) years. The baseline characteristics of this cohort are presented in Table 1. Ultrasound was the most frequently employed imaging modality, performed in 90% of cases, followed by magnetic resonance imaging in 49% of patients. Genetic analyses revealed that among the 145 children, 20 (14%) had TSC1 mutations and 81 (56%) had TSC2 mutations, including 3 children with the contiguous gene syndrome (TSC2/PKD1) that were included in the TSC2 group for subsequent analysis. Of the 145 imaging reports available on record, 114 (79%) revealed abnormal findings suggestive of TSC-associated kidney disease, encompassing angiomyolipomas (61%), cysts (54%), or both (46%).

**Table 1:** Baseline patient characteristics

**Variable**	**Median (IQR) or** ***n*** **(%) (*N* = 145)**
**Median current age**	11.2 (6.8–16.9) years
**Median age at diagnosis**	9 (4–26) months
**Sex (male)**	74 (51%)
**Genetic testing**	
TSC 1	20 (14%)
TSC 2	81 (56%)
No mutations identified	11 (7.6%)
Unknown	33 (23%)
**Family history**	32 (22%)
**Presenting symptoms**	
Neurologic	139 (96%)
Skin lesions	138 (95%)
Delayed development	100 (69%)
Back pain or haematuria	5 (3.4%)
**Kidney imaging modality**	
Ultrasound	130 (90%)
Magnetic resonance imaging	71 (49%)
**Kidney lesions**	
Angiomyolipoma	88 (61%)
Cysts	78 (54%)
Both angiomyolipoma and cysts	67 (46%)
**Treatment**	
mTOR inhibitor therapy	11 (7.6%)
Embolisation	1 (0.7%)

*IQR* interquartile range, *mTOR* mechanistic target of rapamycin, *TSC* tuberous sclerosis complex

### Kidney cysts and/or angiomyolipomas development

Tables 2 and 3 present the hazard of having kidney cysts and angiomyolipomas among children with TSC, respectively. From the multivariate analysis, gender was a significant predictor for kidney cysts occurrence; females had a 50% less chance of developing cysts. Both nutritional status and genetic mutation did not play a significant effect in cysts development; however, both had a significant effect on kidney angiomyolipomas. Gender, nutritional status, and genetic mutation were all significant contributors to angiomyolipoma development in TSC cases in children. Obesity had 2.6 times (*p* = 0.011) significantly more likelihood of developing kidney angiomyolipomas as compared to children with normal BMI (5th–<85th centiles); the risk decreases with subsequent BMI reduction. Like kidney cysts, the hazard of having kidney angiomyolipomas was over twice higher in patients with TSC2 mutation than in those with TSC1 mutation (*p* = 0.029).

**Table 2:** Variables estimate for kidney cysts using Cox proportional hazard regression model

**Outcome variable**	**Explanatory variables**	**B**	**SE (B)**	**HR**	***p*-value **	**95% CI**
Kidney cysts	Gender (female)	−0.688	0.328	0.503	**0.036**	0.264, 0.956
	Genetic mutation (TSC2)	0.775	0.419	2.170	0.064	0.955, 4.933
	BMI < 5th centiles (underweight)	−1.727	1.044	0.178	0.098	0.023, 1.374
	BMI 85th–< 95th centiles (overweight)	−0.079	0.427	0.924	0.854	0.400, 2.134
	BMI > 95th centiles (obesity)	−0.122	0.409	0.885	0.765	0.397, 1.972

*B* coefficients for the predictors in the model, *BMI* body mass index, *CI* confidence intervals, *HR* hazard ratio, *SE (B)* standard error of regression of the coefficient (B)

**Table 3:** Variables estimate for kidney angiomyolipomas using Cox proportional hazard regression model

**Outcome variable**	**Explanatory variables**	**B**	**SE (B)**	**HR**	***p*-value**	**95% CI**
Kidney angiomyolipomas	Gender (female)	−0.683	0.316	0.505	**0.030**	0.272, 0.937
	Genetic mutation (TSC2)	0.943	0.432	2.568	**0.029**	1.101, 5.989
	BMI < 5th centiles (underweight)	−2.388	1.105	0.092	**0.031**	0.011, 0.800
	BMI 85th–< 95th centiles (overweight)	0.415	0.413	1.515	0.314	0.675, 3.401
	BMI > 95th centiles (obesity)	0.938	0.368	2.555	**0.011**	1.243, 5.255

*B* coefficients for the predictors in the model, *BMI* body mass index, *CI* confidence intervals, *HR* hazard ratio, *SE (B)* standard error of regression of the coefficient (B)

### Kidney progression to high-risk angiomyolipomas and CKD

We further analyse kidney progression to CKD and high-risk angiomyolipomas—the two leading causes of mortality in adult TSC cases. Among the 145 children, kidney function was assessed in 105 (72%); however, an eGFR estimate cannot be derived from 24 children due to missing data. Of the remaining 81 children, 10 (12%) developed CKD stages II–V. Overall, 13/145 (9%) children with kidney imaging performed had kidney angiomyolipomas above 3 cm—the cutoff used in published literature and clinical guidelines for representing lesions with an increased risk of acute haemorrhage. Nonetheless, none of our patients experienced haemorrhage.

Six of ten (60%) CKD cases had TSC2 mutations, and none with TSC1 mutations. Notably, only angiomyolipoma above 3 cm appeared to be an independent predictor of CKD development in the univariate regression model, with a statistically significant regression coefficient (OR 15.333; *p* = 0.007) (Table 4). This implies a 15-fold greater risk of developing CKD if an individual has an angiomyolipoma above 3 cm. With such, we aim to explore further the factors associated with high-risk angiomyolipomas.

**Table 4:** Unadjusted logistic regression model for chronic kidney disease

**Parameters**	**Unadjusted ORs**	**95% CI**	***p*-value**^a^
Current age	1.003	0.990–1.017	0.627
Age at first presentation of angiomyolipoma	0.990	0.973–1.008	0.283
Age at first presentation of cyst	0.989	0.973–1.005	0.167
Gender	2.900	0.484–17.377	0.244
Kidney cysts	1.161	0.193–6.983	0.870
Kidney angiomyolipomas	0.441	0.080–2.443	0.349
Overweight / obesity^b^	1.765	0.320–9.732	0.514
Angiomyolipomas > 3 cm	15.333	2.115 –111.184	**0.007**

*CI* confidence intervals, *OR* odds ratios

^a^*p-value* is obtained with univariate logistic regression

^b^Overweight / obesity: BMI ≥ 85th percentiles

Of the 13 children with high-risk angiomyolipomas, 8 (62%) had TSC2 mutation, and 1 (7.7%) had TSC1 mutation, while others had NMI or unknown genetics. Logistic regression analysis suggests the odds of having high-risk kidney lesions in patients with TSC2 mutations is 1.2 times higher than those with TSC1 mutations, although this association was not significant (OR 1.172; *p* = 0.891) (Table 5).

**Table 5:** Unadjusted and adjusted logistic regression model for angiomyolipomas > 3 cm

**Parameters**	**Unadjusted ORs**	**95% CI**	***p*-value**^a^	**Adjusted ORs**^b^	**95% CI**	***p*-value**^c^
Current age	1.021	1.004–1.037	**0.014**	1.015	1.004–1.026	**0.008**
Age at first presentation of angiomyolipomas	0.990	0.973–1.007	0.249			
Gender (female)	2.856	0.656–12.441	0.162			
Genetic mutation (TSC2)	1.172	0.121–11.341	0.891			
Overweight/obesity^d^	7.520	1.881–30.068	**0.004**	7.129	1.940–26.202	**0.003**

*CI* confidence intervals, *OR* odds ratios

^a^*p-value* obtained with univariate logistic regression

^b^Adjusted for factors with univariate *p-value* < 0.05

^c^*p-value* obtained with multivariate logistic regression

^d^Overweight/obesity: BMI ≥ 85th centiles

On univariate logistic regression analysis, only current age and being overweight/obese were independently associated with increased odds of having angiomyolipomas above 3 cm in children with TSC. Our multivariate regression modelling showed that current age (aOR 1.015, *p* = 0.008) and being overweight/obese (aOR 7.129, *p* = 0.003) were significantly associated with high-risk angiomyolipomas. The Nagelkerke R^2^ indicated that this adjusted model accounts for 22% of the variability in angiomyolipomas above 3 cm (*p* < 0.001).

## Discussion

Tuberous sclerosis is a heterogeneous disorder that affects multiple organs, including the kidneys. The awareness of the predictors of TSC-associated kidney disease is crucial for early identification and management to prevent progression to life-threatening complications, such as CKD and acute haemorrhagic episodes [3].

Our study revealed a significantly lower probability of females developing kidney cysts compared to males (HR 0.503, *p* = 0.036). Although TSC2 mutation doubled the risk of developing cysts than TSC1, statistical significance was not reached. Building on prior research by Gallo-Bernal et al. [12], we identified a 2.6-fold higher likelihood of developing kidney angiomyolipoma in patients with TSC2 mutation, reinforcing the role of TSC genotype as a predictor for kidney involvement [13]. On multivariate analysis, females and being underweight had lower risk, while obesity was associated with a higher risk of angiomyolipomas occurrences. Our Cox proportional hazard regression model addressed the conflicting findings in prior studies on gender differences [4, 8, 14, 15], showing that females had half the risk of developing angiomyolipomas than males over time. This aligns with studies suggesting the protective effects of oestrogen on kidneys, promoting antioxidant and anti-inflammatory actions and thereby slowing the progression of CKD [16] and kidney cancer [17]. In contrary, other studies have elucidated that angiomyolipomas were predominantly found in female patients due to the influence of oestrogen and progesterone on angiomyolipoma tumour receptors [9], promoting greater growth propensity [12, 18]. To clarify these contradictory findings, we explored research on lymphangioleiomyomatosis, predominantly observed in reproductive-aged woman with TSC, known to have commonalities with angiomyolipomas, such as a shared origin from epithelioid cell and similar genetic and biological characteristics [19]. The review by Prizant and Hammes [20] on lymphangioleiomyomatosis suggested its development and progression are dependent on oestrogen signalling, with higher progression rates during elevated oestrogen levels (e.g. pregnancy or exogenous oestrogen consumption), and lower rates during oestrogen decline (e.g. menopause). Considering that the median age of our study participants is 11.2 years, possibly in a pre-pubertal stage with relatively low oestrogen levels, this may explain the difference between our study and previously published studies that suggested females were at higher risk since these previous studies included mainly adult participants, hence higher oestrogen levels [8, 14]. Drawing parallels, the relatively higher oestrogen level also observed in obese patients could potentially explain the increased hazard in the development and progression of kidney angiomyolipomas in our patients. This aligns with the findings of Sun et al. [21] that noted obesity as a risk factor for kidney cancer, promoting a series of secondary pathologies, such as insulin resistance and abnormal adipokines expression occurs, leading to a persistent inflammatory state that contributes to cancerous development [10, 22]. In non-TSC adult studies, the risk of kidney cancer increased by 1.06 times for every unit increase in BMI [10]. To the best of our knowledge, our study is the first to analyse BMI as a predictor for TSC-associated kidney angiomyolipomas development in children. We found that the hazard increases in obese patients and decreases in underweight individuals. This conflicts with the findings of Liu and colleagues [22] that suggested sporadic, fat-poor angiomyolipomas to be unrelated to obesity, though this was in non-TSC adult patients and of relatively smaller sample size. We hypothesised that persistent inflammation and elevated oestrogen levels associated with obesity underlie angiomyolipoma growth in TSC patients [16, 17, 21, 22], drawing parallels with studies on the upregulating effect of oestrogen on the influence of obesity on kidney [21] and colorectal cancer [23]. Another hypothesis explores the role of excess nutrients in obese patients in the dysregulation of the mTOR complex. An in vitro study involving obese rats revealed that obesity triggers chronic excessive activation of mTOR activity in multiple tissues [24], consistent with the broader concept of nutritional sufficiency in regulating mTOR signalling activity [25]. This supports our findings that being underweight serves as a protective factor for both kidney cysts and angiomyolipomas, with statistical significance observed specifically in kidney angiomyolipomas. Further research is warranted to unravel these complex interactions.

TSC-related kidney complications, including CKD and acute haemorrhage, significantly contribute to TSC patients’ mortality [3]. Prior studies have reported a higher morbidity rate of CKD stages III–V in TSC patients compared to the general population by their mid-50s (41 vs. 3%) [7]; however, this study only included TSC patients above 18 years. A follow-up of TSC individuals in Belgium found that 35% (28/80) had CKD stages II–V, with six cases of kidney failure occurring at a median age of 42 years [5]. In our study, we found that 10/81 (12%) children with available kidney function had CKD above stage 2, and only two (2.5%) had an eGFR below 60 mL/min/1.73m^2^ (stages III–V). It should be emphasised that estimating eGFR in children is challenging given the varying cutoffs for determining CKD, particularly those under 2 years. Consequently, our study might have potentially underdiagnosed cases with CKD. Despite the small number of CKD cases (*n* = 10), we found a significant association between CKD and angiomyolipomas larger than 3 cm (*p* = 0.007). This suggests angiomyolipomas exceeding 3 cm may contribute to CKD, carrying a 15-fold greater risk compared to angiomyolipomas under 3 cm.

The threshold size for classifying an angiomyolipoma lesion at high risk of haemorrhage varies between recommendations, ranging from 3 to 4 cm [26–29]. In our study, we used the 3 cm cutoff to encompass more cases and provide evidence of risk factors for angiomyolipomas beyond 3 cm, aiming to enhance awareness and care. UK funding guidelines indicate that children with angiomyolipomas above 3 cm are eligible for everolimus treatment [30]; conversely, the updated 2021 TSC guidelines advised everolimus use for asymptomatic angiomyolipomas above 4 cm or intra-lesional aneurysms over 5 mm [28]. Lesions above these sizes are deemed at an increased risk of acute haemorrhage; there were no haemorrhage among the 13 children (9%) with high-risk angiomyolipomas in our study.

Interestingly, the duration of time since the initial detection of angiomyolipoma does not show any association with angiomyolipomas larger than 3 cm. Meanwhile, the current age of the participants and being overweight/obese are associated with high-risk angiomyolipoma lesions. This indicates that overweight or obese individuals with TSC are more susceptible to developing kidney angiomyolipomas, especially high-risk angiomyolipomas. These findings underscore the importance of regularly monitoring children with reduced eGFR or those who are overweight/obese to track angiomyolipoma development. Current recommendations suggest magnetic resonance imaging and alternating with ultrasound for kidney surveillance every 1 to 3 years in TSC patients without kidney lesions [26, 28]. Patients with stable angiomyolipomas below 3 cm should be monitored annually, but those with atypical or fast-growing lesions should be assessed at shorter intervals due to the risk of haemorrhage [12]. In cases of angiomyolipoma haemorrhage, the first-line management is embolisation and corticosteroids rather than nephrectomy, as it offers the advantage of preserving kidney functions [31, 32].

In addition to kidney imaging for monitoring, regular assessment of ambulatory blood pressure is advised for children with TSC, as emphasised by Vargiami and colleagues [33]. Their work also highlights that increased eGFR could potentially serve as an early marker of hypertensive kidney dysfunction. Besides medical management, a holistic approach to TSC care involves a multidisciplinary team of professionals to provide coordinated care, including mental well-being for patients and caregivers.

Fang et al. [34] highlighted that the ketogenic diet is characterised by a low-carbohydrate and fat-rich profile, as an effective and safe treatment for TSC children with intractable epilepsy and cognitive impairment. Their study demonstrates over 50% reduction in seizures, and more than 70% of children showed improvements in cognitive function and behaviour. However, prior studies on TSC rat models demonstrated a potential promotion of kidney tumour growth with long-term ketogenic diet [35]. Additionally, a case series reported kidney angiomyolipoma growth in two out of five TSC patients, both children, while on the ketogenic diet [36]. Despite the inclusion of some children managed with the ketogenic diet in our study, unfortunately, we lacked necessary data to comprehend its impact on the growth of kidney lesions.

This study has several limitations. Firstly, it adopts a retrospective design, and although the overall cohort is large for a paediatric study of a rare disease, some of the individual sub-groups are small, limiting the power to detect a statistically significant effect. Secondly, kidney involvement was determined by assessing kidney imaging, with the potential for inter-operator variability. The availability of eGFR data in only 81 patients raises the possibility of overestimating the true incidence of CKD. Three children had TSC2/PKD1 contiguous gene deletion syndrome, which may provide a slight bias to the results in terms of overall CKD risk and kidney cyst burden. Despite these limitations, this study has the strength of identifying factors associated with the development of kidney lesions and subsequent complications, including the influence of nutritional status, which has, to our best knowledge, not been extensively studied before in children. Additionally, the study highlights this novel finding which allows us to postulate on the potential modifying effect of oestrogen on the relationship between obesity and angiomyolipoma development in children with TSC. Future studies can perform longitudinal follow-up studies to provide insights into disease progression and long-term kidney outcomes. Considering our findings, further studies should investigate the potential role of nutrition in kidney disease, incorporating nutritional status as a factor in the analysis. Many TSC patients also have autism and may adhere to a restricted diet. A detailed recording of food intake, including protein consumption and salt intake, can be valuable in uncovering potential connections between diet, disease progression, and kidney complications.

In conclusion, the kidneys are typically affected in children with TSC. This study adds an understanding of the factors that predict kidney involvement and the subsequent progression into CKD and high-risk angiomyolipomas. Our findings emphasise the importance of kidney surveillance in caring for children with TSC, including routine kidney imaging and kidney function monitoring. Although rare, complications can still occur in children, highlighting the implementation of kidney prevention strategies in childhood. Overweight/obese children or those with CKD should be frequently monitored for high-risk angiomyolipomas based on the novel findings from this study.

## Electronic supplementary material

Below is the link to the electronic supplementary material.

Supplementary file1

## Abbreviations

aOR: Adjusted odds ratio
BMI: Body mass index
CKD: Chronic kidney disease
eGFR: Estimated glomerular filtration rate
HR: Hazard ratio
mTOR: Mechanistic target of rapamycin
TSC: Tuberous sclerosis complex
