# Supplementary figures and images for: Nutritional status as a predictive factor for paediatric tuberous sclerosis complex-associated kidney angiomyolipomas: a retrospective analysis
Source: Eur J Pediatr. 2024 Mar 14;183(6):2563–70. doi: 10.1007/s00431-024-05520-8 (PMC11098920; doi:10.1007/s00431-024-05520-8)

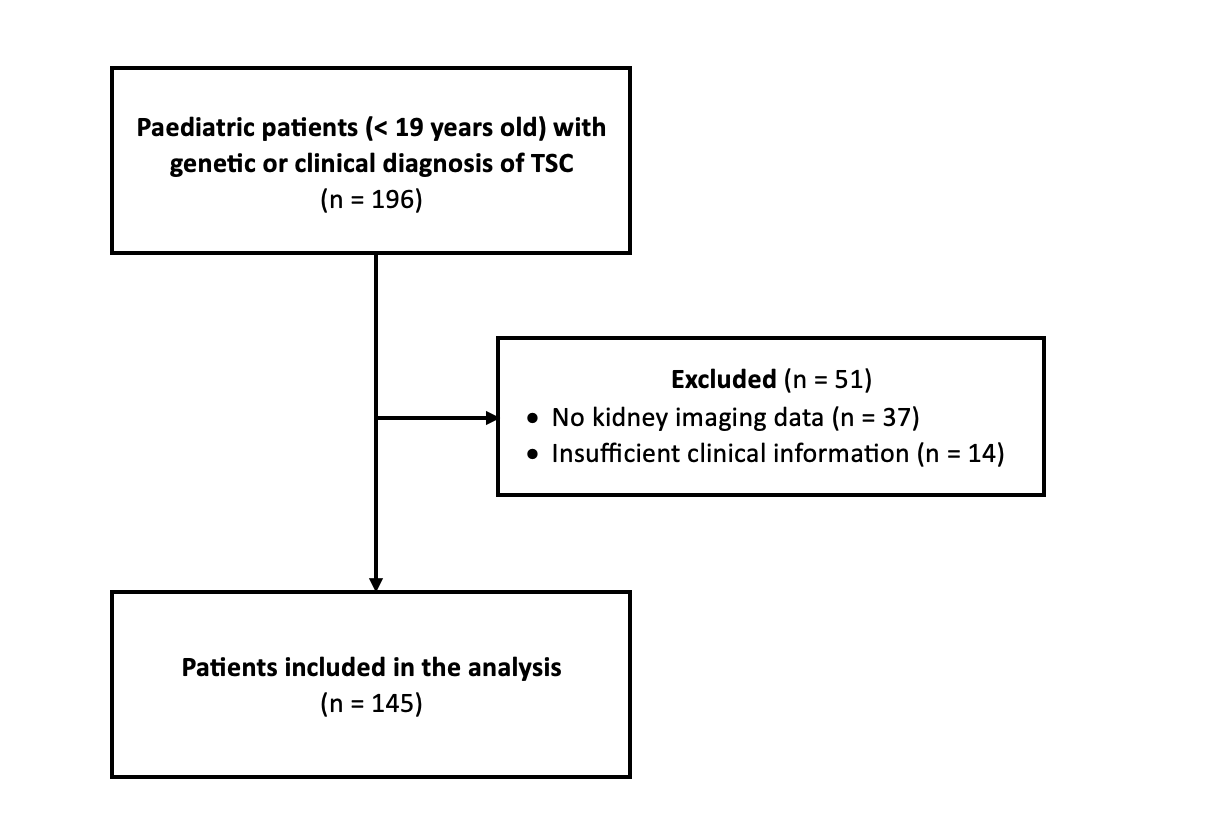

Supplement: Supplementary file 1 — Supplementary file1 [file 431_2024_5520_MOESM1_ESM.tiff]
